# Preparation of Carboxymethyl Cellulose-Based Macroporous Adsorbent by Eco-Friendly Pickering-MIPEs Template for Fast Removal of Pb^2+^ and Cd^2+^

**DOI:** 10.3389/fchem.2019.00603

**Published:** 2019-09-10

**Authors:** Feng Wang, Yongfeng Zhu, Hui Xu, Aiqin Wang

**Affiliations:** ^1^Key Laboratory of Clay Mineral Applied Research of Gansu Province, Center of Eco-Material and Green Chemistry, Lanzhou Institute of Chemical Physics, Chinese Academy of Sciences, Lanzhou, China; ^2^College of Petroleum and Chemical Engineering, Beibu Gulf University, Qinzhou, China; ^3^Department of Chemical Engineering, College of Petrochemical Engineering, Lanzhou University of Technology, Lanzhou, China

**Keywords:** macroporous materials, Pickering emulsions, montmorillonite, adsorption, heavy metal

## Abstract

Recently, Pickering high internal phase emulsions (Pickering HIPEs) have been widely used to fabricate macroporous materials. However, the high usage of poisonous organic solvent in HIPEs not only greatly increases the cost but also is harmful to human health and environment, which leads to limited large-scale applications. In this study, we prepared a novel monolithic macroporous material of carboxymethyl cellulose-*g*-poly(acrylamide)/montmorillonite (CMC-*g*-PAM/MMT) by the free radical polymerization *via* oil-in-water Pickering medium internal phase emulsions (Pickering MIPEs), which used the non-toxic and eco-friendly flaxseed oil as continuous phase, MMT, and Tween-20 (T-20) as stabilizer. The pore structure of the resulting macroporous materials could be tuned easily by adjusting the content of MMT, co-surfactant T-20, and the oil phase volume fraction. The maximal adsorption capacities of the prepared macroporous material for Pb^2+^ and Cd^2+^ were 456.05 and 278.11 mg/g, respectively, and the adsorption equilibrium can be reached within 30 min. Otherwise, the macroporous monolith exhibited excellent reusability through five adsorption–desorption cycles. Thus, the eco-friendly Pickering-MIPEs is a potential alternative method to be used to fabricate multi-porous adsorption materials for environmental applications.

**Graphical Abstract:**
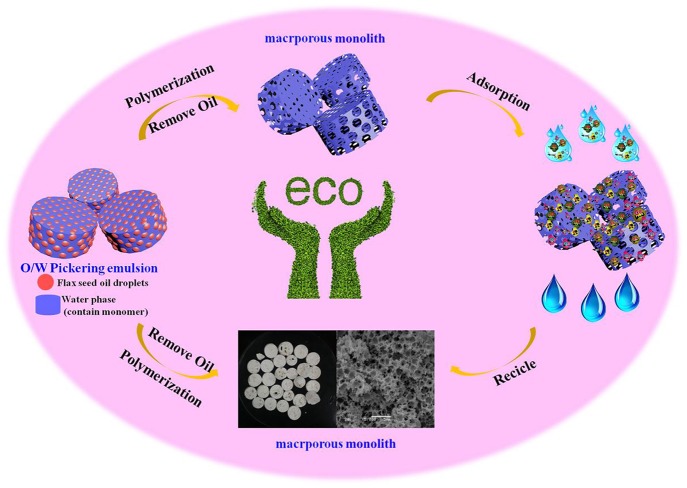
Preparation of macroporous adsorbent from Pickering MIPEs for removal of heavy metal.

## Introduction

Recently, macroporous materials with high porosity and good interconnectivity have attracted much attention in many fields, such as catalysts (Pierre et al., 2006; Zhang et al., 2008; Chan-Thaw et al., 2010; Pulko et al., 2010; Kovačič et al., 2014; Wang et al., 2014), tissue engineering scaffolds (Busby et al., 2001; Zhang et al., 2005; Hu et al., 2014; Viswanathan et al., 2015), ion exchange columns (Zhao et al., 2007; Alexandratos, 2008), electrode materials for energy storage (Liu et al., 2008; Kou et al., 2011), and water purification (Yu et al., 2015; Zhu et al., 2016c; Wu et al., 2017). Thus, many methods have been used to prepare macroporous materials. Among these technologies, the emulsion template may be the most effective method to prepare the macroporous polymer with extraordinary advantages, including a well-defined pore structure, a controllable pore size, as well as inherent high porosity (Xu et al., 2015). The most well-known emulsion template technology is the high internal phase emulsions (HIPEs), which, with an internal phase volume of >74% and the prepared macroporous polymers, are also termed poly-HIPEs.

Conventional HIPEs are commonly stabilized with a large amount of surfactants (5–50 vol % with respect to the continuous phase) (Zheng et al., 2014). Inevitably, the heavy use of these surfactants significantly increases the high cost of materials (Wu et al., 2012). Thus, more and more studies involving HIPEs have focused on the Pickering HIPEs, which employ a small amount of solid particles substituting surfactants partially or totally to stabilize emulsions (Binks and Lumsdon, 1999). In addition, the macroporous poly-HIPEs created from Pickering HIPEs often have improved mechanical strength (Silverstein, 2014), additional features, and functions, due to the introduction of solid particles, such as magnetic property, photocatalytic performance, and so on (Vílchez et al., 2011).

Despite the fact that macroporous poly-HIPEs prepared from Pickering HIPEs have these excellent properties, some problems still need to be solved. Firstly, the macroporous poly-HIPEs prepared from Pickering-HIPEs, which are only stabilized by solid particles, often have closed-cell voids and super porous structures with void sizes of 200–700 μm (Ikem et al., 2010a,b, 2011). The weak permeability and the low surface area limit the application of the macroporous materials in many fields, especially in water treatment. Secondly, it also should be noted that oil/water (O/W) HIPEs often require more than 74% of organic solvent (such as toluene, *n*-hexane, *p*-xylene, liquid paraffin, and so on) in disperse phase. The wide use of these organic solvents greatly increases the cost as well as the threat to human health and environment. Thus, discovery of a new strategy, which can reduce the oil phase volume and replace the poisonous organic solvent with eco-friendly natural oil for preparation of the macroporous materials with interconnected pore structure *via* emulsion template is very meaningful.

Compared with HIPEs, medium internal phase emulsions (MIPEs, V < 74%) require less organic solvents and decrease the diameter of the emulsion droplet, which is beneficial in increasing material surface area. Ikem et al. (2010b, 2011) have reported that introducing a small amount of surfactant (~5% of the continuous phase volume) to stabilize Pickering HIPEs cooperatively with particles before polymerization will reduce the void sizes of the poly-Pickering HIPEs and results in materials with large pore throats and permeability. Otherwise, the permeability and connectivity of the poly-MIPEs can be adjusted by changing the content of nanoparticles or introducing an appropriate amount of surfactant (Wang et al., 2017a,b). According to the preceding discussion, in this study, we reported a new strategy for fabrication of macroporous material from Pickering-MIPEs, which is stabilized by montmorillonite (MMT) together with nonionic surfactant, and non-toxic low-cost flaxseed oil was used as emulsion in disperse phase. MMT, being a natural silicate clay mineral with abundant -OH groups and negatively charged, is highly hydrophilic; thus, MMT silicate clay can be used to stabilize O/W emulsion without any further modification to regulate its wettability (Shen et al., 2006; Dong et al., 2014). To the best of our knowledge, most of the present research on clay mineral-stabilized emulsion focus on the stability of emulsion or on the preparation of microspheres with Pickering emulsion polymerization. Studies involving preparation of highly macroporous polymer monoliths *via* clay mineral-stabilized Pickering emulsion template are rare. T-20 used in this study was a co-stabilizer emulsion and contributes to formation materials with connected pores. In this research, the pore structure of the prepared macroporous monolith was studied by tuning of MMT and Tween-20 (T-20) concentration and O/W ratio. The potential practical application of the macroporous polymer monoliths in the removal of heavy metal ions (Pb^2+^ and Cd^2+^) from aqueous solution was also explored (Graphical Abstract).

## Experimental Section

### Materials

Sodium carboxymethylcellulose (CMC, chemical pure, with a viscosity of 300–800 mPa·s), ammonium persulfate (APS, analytical pure), and Tween-20 (T-20, analytical pure) were purchased from BASF Corporation. Na-MMT clay mineral with a cation exchange capacity (CEC) of 90 mEq/100 g was purchased from Southern Clay Products Inc. Flaxseed oil (FO, food grade) was purchased from the Lanzhou Yong Fan Shang Mao company (Lanzhou, China). Acrylamide (AM, chemical pure) was purchased from Shanpu Chemical Factory (Shanghai, China) and used without further treatment. *N, N*′-methylenebisacrylamide (MBA, chemical pure) was received from Yuan fan additives plant (Shanghai, China). Other reagents were all analytical pure and all solutions were prepared with distilled water.

### Preparation of Pickering Emulsion

In order to guarantee that MMT dispersed completely in water, the MMT was initially prepared as a suspension with a certain concentration. Briefly, 10 or 15 g of MMT was added into 100 mL of deionized water and sonicated with BILON-650Y Sonifier for 30 min. In the preparation process of the Pickering emulsion, 10 mL of water containing a certain amount of MMT and an appropriate amount of T-20 was stirred at 300 rpm for 2 h in the boiling flask-3-neck in the continuous phase. Then, a determined volume of flaxseed oil was added into the continuous phase and emulsified with a GJD-B12K homogenizer at 11,000 rpm for 5 min.

### Preparation of the Macroporous Monolith

The macroporous monolith was prepared *via* the free radical polymerization in the Pickering emulsion template. Firstly, a Pickering emulsion containing 0.05 g of CMC and 0.23 g of MBA was prepared according to the section *Preparation of Pickering Emulsion*. Subsequently, 2.13 g (30 mmol) of AM and 138 mg of APS were added into the as-prepared emulsion and rapidly stirred (11,000 rpm) for 1 min. Later, the emulsion was transferred into 10-mL centrifuge tubes and immersed in a 65°C water bath for 24 h to polymerize. After that, the obtained polymerization product was sectioned and washed with acetone by Soxhlet extraction for 12 h and then immersed into 0.5 mol/L NaOH aqueous alcohol solution (V_water_/V_alcohol_ = 3/7) for 24 h to transfer the amide group to carboxyl. The redundant NaOH was washed with alcohol–water solution repetitively. Finally, the macroporous polymer was dried at 60°C for 4 h and named CMC-*g*-PAM/MMT. The feed compositions and the corresponding pore parameters of all the macroporous monoliths are listed in Table 1.

**Table 1:** Feed composition of the macroporous monolith and their corresponding average pore size (D) and surface area (A).

**Codes**	**T-20 (%)**	**MMT (%)**	**Oil (%)**	***D*_**m(macro)**_ (μm)**	***D*_**m(pore throat)**_ (μm)**	***A*_**(macro)**_ (μm^**2**^)**	***A*_**(pore throat)**_ (μm^**2**^)**
Sample 1	2	3	50	1.52	0.44	307.52	62.47
Sample 2	2	5	50	3.37	–	379.71	–
Sample 3	2	7	50	2.75	–	394.44	–
Sample 4	2	9	50	1.89	–	439.04	–
Sample 5	3	5	50	1.43	0.52	418.88	112.49
Sample 6	4	5	50	1.38	0.40	409.48	125.29
Sample 7	5	5	50	1.25	0.37	325.24	207.91
Sample 8	6	5	50	1.26	0.37	431.70	230.26
Sample 9	4	5	40	1.57	0.47	396.51	255.01
Sample 10	4	5	30	1.71	0.41	293.71	140.29
Sample 11	4	5	20	2.13	0.53	248.77	33.64

### Characterization

The droplet diameters were estimated by counting 200 droplets by using Image Pro Plus as a software tool. The morphologies of the samples were characterized by a field emission scanning electron microscope (SEM, JSM-6701F, JEOL) after coating the samples with gold film. Photographs of Pickering-MIPEs were recorded by iPhone. The infrared (IR) spectra were recorded on a Nicolet NEXUS FTIR spectrometer in the range of 4,000–400 cm^−1^ using KBr pellets.

### Batch Adsorption Studies

The adsorption experiments were performed by immersing 20-mg macroporous monolith into 25 mL Pb^2+^ or Cd^2+^ solutions and shocking in a thermostatic shaker (THZ-98A) at 120 rpm at 30°C for a given time to reach adsorption equilibrium. After the adsorption, the concentrations of Pb^2+^ and Cd^2+^ solutions were analyzed by a UV–visible spectrophotometer (UV-3010, HITACHI). The adsorption capacity *q*_e_ (mg/g) of the macroporous monolithic adsorbents was calculated according to the following equation:

(1) $$q_{\text{e}}\;=\;(C_{0}-C_{\text{e}})V/m$$

where *C*_0_ and *C*_e_ are the initial and the final concentration of the Pb^2+^ and Cd^2+^, *V* is the volume of the Pb^2+^ and Cd^2+^ solution, and *m* is the adsorbent dosage.

The required pH of solution was adjusted by 0.1 mol/L HCl or NaOH solutions. The adsorption isotherms were conducted by adding adsorbents (Sample 6) into Pb^2+^ and Cd^2+^ solutions for 2 h with concentration in the 100–600 mg/L range, and the control factors of the adsorption process were evaluated with the Langmuir and the Freundlich isotherm model. The adsorption kinetics were determined with 400 mg/L Pb^2+^ and Cd^2+^ solutions by varying the adsorption time from 5 to 120 min, and the pseudo-first-order equation and pseudo-second-order equation were carried out to describe the adsorption process. All adsorption experiments were repeated thrice to guarantee the accuracy of the obtained data.

The desorption studies were performed to evaluate the recyclability of the macroporous adsorbent. After the adsorption process, the adsorbents were separated and immersed into 30 mL of HCl solution (0.5 mol/L) for 2 h to desorb and then activated with 0.5 mol/L NaOH solution for 2 h. After that, the adsorbents were washed with distilled water till neutral pH and used in the next adsorption process. The adsorption–desorption cycle was performed five times.

## Results and Discussion

### Preparation and Structure Characteristics of CMC-G-PAM/MMT

#### Formation of Pickering Emulsion and the CMC-G-PAM/MMT

**Figure 1:**
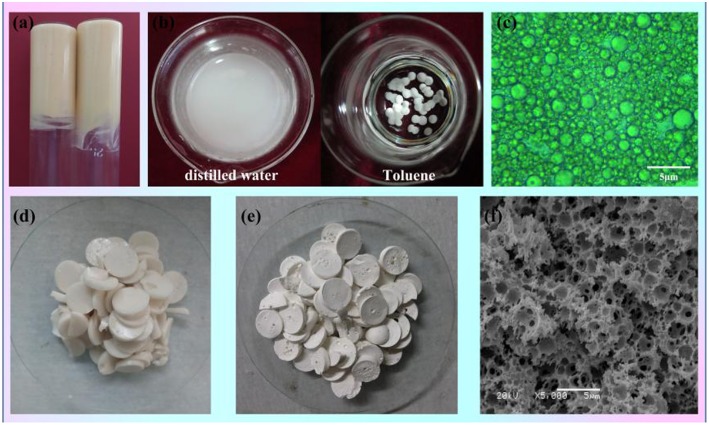
**(a)** Digital photographs of the prepared Pickering MIPEs (MMT, 5%;T-20, 4%; internal phase volume, 50%). **(b)** Testing the type of emulsion by dropwise adding the emulsion droplet into the distilled water (DI) or toluene. **(c)** Optical images of the Pickering MIPEs. **(d)** Digital photographs of CMC-*g*-PAM/MMT before Soxhlet extraction. **(e)** Digital photographs and **(f)** SEM of porous CMC-*g*-PAM/MMT.

As shown in Figure 1a, the paste-like concentrated Pickering emulsion (Figure 1a) was prepared by adding dropwise flaxseed oil into the aqueous phase, with MMT and T-20 as the stabilizer. Firstly, the MMT interacted with Tween-20 and formed the surfactant-coated MMT, and then the coated MMT clay particle combined with each other in the water phase to create a 3D network called the “cards house” aggregation model. Because the flaxseed oil is yellow, the Pickering MIPEs were canary yellow. The emulsions can stay in the reversed glass tube without any flow, suggesting that the formed emulsion is a typical gel emulsion and the oil drop is closely packed, which is verified by its optical micrograph as shown in Figure 1c. It can also be observed that the size of oil drops varies from around hundreds of nanometers to several micrometers. The type of emulsion was detected by the pendant-drop method; as presented in Figure 1b, the emulsion droplet dispersed into the distilled water but remained round in toluene, indicating that the emulsion is an O/W emulsion.

The porous monoliths of CMC-*g*-PAM/MMT were synthesized by free radical polymerization with APS as the initiator. The sulfate anion radicals formed from the decomposition of APS under 65°C, and the -OH groups of CMC were activated and formed the macro-radicals. Then, the macro-radicals triggered the double bonds of AM and *in situ* initiated the grafting reaction of the vinyl monomers onto the CMC in the presence of the cross-linker MBA. Once the polymerization reaction ended, the prepared wet monoliths were cut into pieces (Figure 1d) and Soxhlet extracted by acetone to remove off the oil phase, and the end product of white monoliths were finally obtained after drying (Figure 1e). The macroporous polymer monoliths will finally be used as adsorbent to remove heavy metal ions from water; thus, the amide group of the monoliths must be hydrolyzed with NaOH solution to generate -COO-, based on the fact that -COO- groups are easier to combine with heavy metal ions than the acylamino. Because of the hydrogen-bond interaction between polar groups (Zhu et al., 2016a), the prepared monoliths can swell rapidly in the water and shrink during the drying process. In order to avoid pore collapse, hydrolysis was conducted in a NaOH alcohol water mixture (V_ethanol_/V_water_ = 7/3) (Zhu et al., 2016c). Then, the obtained CMC-*g*-PAM/MMT was dehydrated by Soxhlet extraction with acetone again and dried in an oven at 40°C. A photograph of the final resulting CMC-*g*-PAM/MMT was shown in Figure 1e. Figure 1f showed the CMC-*g*-PAM/MMT with a well-interconnected macroporous texture. It could be observed that both the macropores and highly interconnected small pores coexisted in the resulting material. The large cell pores were generated by removal of the oil droplets, and the pore throat derives from the thinnest points of the two neighboring oil drops attributed to the shrinkage during polymerization (Zhu et al., 2016c). The introduction of MMT not only stabilized the emulsion but also participated in polymerization to dramatically improve the performance and mechanical stability of the polymer-based composite (Schexnailder and Schmidt, 2009; Shibayama, 2012).

#### Effect of MMT Concentration on the Macroporous Structure

**Figure 2:**
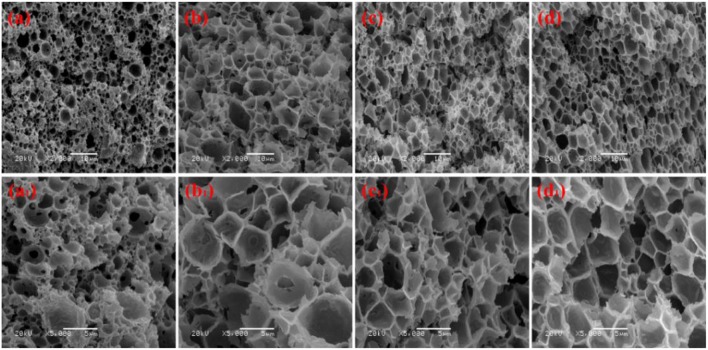
SEM of macroporous CMC-*g*-PAM/MMT with different MMT. Three percentage (**a, a**_**1**_, Sample 1); 5% (**b, b**_**1**_, Sample 2); 7% (**c, c**_**1**_, Sample 3); 9% (**d, d**_**1**_, Sample 4).

We prepared a series of macroporous monoliths from Pickering emulsion, which stabilized with different amounts of MMT particles from 3 to 9% according to the volume of disperse phase. As shown in Figure 2, the enrichment of macrospores can be observed in the matrix of the monoliths. Besides, the surface morphologies of the monolithic materials changed obviously with increasing MMT content from 3 to 9%. The average pore sizes of the prepared monoliths were 1.52, 3.37, 2.75, and 1.89 μm, respectively, corresponding to MMT content that varied from 3, 5, 7, and 9% (Table 1). In the case of 3% MMT, T-20 may play a dominant role for stabilizing the emulsion. When the content of MMT increased to 5%, most of the MMT may be coated with T-20 and then fixed at the O/W interface (Ye et al., 2018). The large size of the coated MMT can only stabilize the droplet larger than T-20; thus, the pore size of macroporous monoliths increased obviously. It was worthy to note that the pore throats existed obviously when 3% MMT was used, but disappeared while increasing the amount of MMT to 5%. This phenomenon has also been found in Pickering emulsions stabilized by other solid particles (Yi et al., 2016), indicating that the pore throats can only form at a moderate ratio of MMT and Tween-20. With an MMT content beyond 5%, the larger interfacial area of emulsion will be stabilized and thus the droplet size decreased. The macropore and pore throat void size distribution of the macroporous monolith with different MMT concentrations was shown in Figure S1.

**Figure 3:**
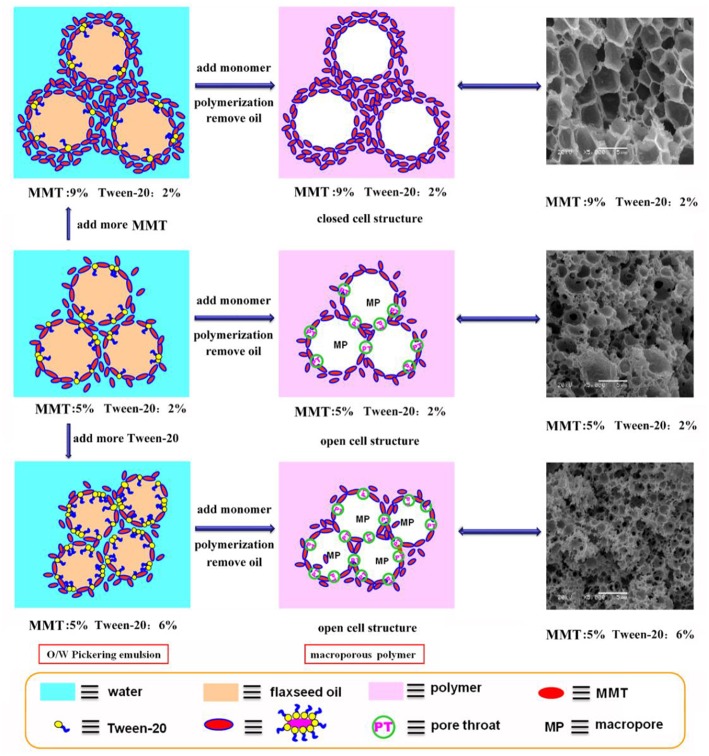
Mechanism for the formation of CMC-*g*-PAM/MMT with closed-cell structure or open-cell structure by adding the MMT and T-20.

In general, the increase in particle content will result in smaller droplet size and close packing of droplets, thus leading to more open pores during polymerization. However, as shown in Figure 2, the porous monoliths had a closed pore structure when the amount of the MMT increased from 5 to 9% (Samples 2, 3, and 4). The corresponding possible mechanism was presented in Figure 3. Once the content of MMT increased, the MMT particles packed on the O/W interface around the emulsions closely to form a solid barrier and prevent coalescence (Horozov, 2008). The increase in MMT will lead the aggregation of solid particles at the O/W interface to form a thicker particle layer, which results in a difficult fracture of polymerized film between two adjacent droplets. Thus, it is difficult to obtain monoliths with a desired interconnected structure when a high concentration of the stabilizer is used. These similar results have also been reported by Pickering emulsions stabilized by other solid particles (Wong et al., 2011; Zou et al., 2013b).

#### Effect of Surfactant Concentration on the Macroporous Structure

**Figure 4:**
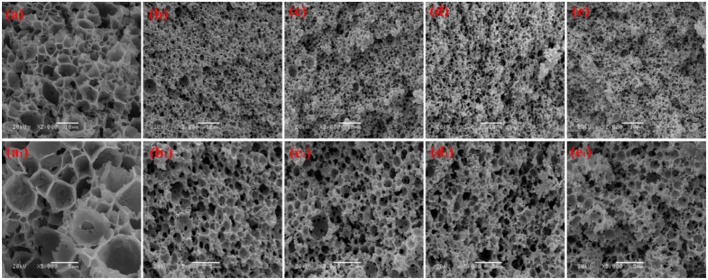
SEM of the macroporous CMC-*g*-PAM/MMT monolith with different Tween-20. **(a, a**_**1**_**)** 2% (Sample 2); **(b, b**_**1**_**)** 3% (Sample 5); **(c, c**_**1**_**)** 4% (Sample 6); **(d, d**_**1**_**)** 5% (Sample 7); **(e, e**_**1**_**)** 6% (Sample 8).

Co-surfactant concentration is also an important parameter for tuning the porosity and interconnectivity of macroporous materials prepared from Pickering emulsion template (Zhang et al., 2009, 2011; Zhu et al., 2010). The increase in co-surfactant concentration will decrease the interfacial tension and contribute to form the interconnected pore's structure simultaneously (Viswanathan et al., 2014). We therefore explored the effect of T-20 content on the microstructure of the macroporous monolith with internal phase volumes at 50% for all the starting emulsions; the SEM images were shown in Figure 4. When T-20 increased from 2% (Sample 2) to 3% (Sample 5), the average diameter of the macropore decreased dramatically from 3.37 to 1.43 μm (Table 1). Further increasing T-20 content from 3% (Sample 5) to 6% (Sample 8), it could be observed that the size of the macropore decreased, the number of pore throats per pore structure increased, and the wall thickness of the polymer layer got thin. Moreover, the surface areas of macropores and the pore throats were all increased as the T-20 content increases (Table 1). When T-20 content increased to 6%, a thin-wall macroporous material with perfect porosity was obtained. It is because more surfactant micelles remained at the O/W interface as T-20 content increases, which contributed to reducing the droplet size of Pickering MIPEs and resulted in forming the interconnected pores (Zou et al., 2013a). The corresponding possible mechanism was shown in Figure 3. The macropore size and the pore throat void size distribution of the macroporous monolith with various T-20 contentions were presented in Figure S1.

#### Effect of Volume Fraction of Disperse Phase on the Macroporous Structure

**Figure 5:**
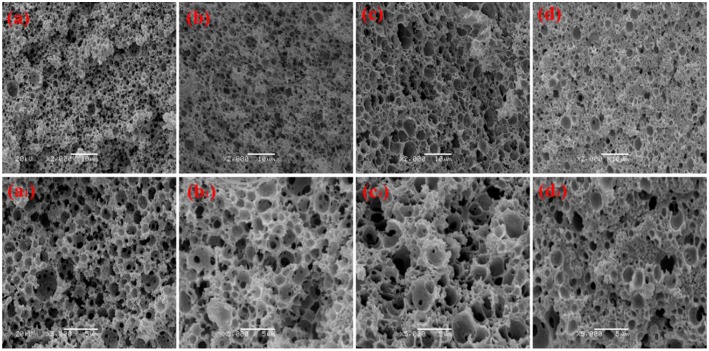
Effect of oil phase volume fraction for the CMC-*g*-PAM/MMT **(a, a**_**1**_**)** 50% (Sample 6); **(b, b**_**1**_**)** 40% (Sample 9); **(c, c**_**1**_**)** 30% (Sample 10); **(d, d**_**1**_**)** 20% (Sample 11). All of the prepared samples prepared with 5% of MMT and 4% of T-20.

The pore size and interconnectivity of the macroporous materials are influenced significantly by the internal phase fraction. The macroporous materials prepared from different oil phase fractions of 50, 40, 30, and 20 vol % are presented in Figure 5. With the oil phase volume fraction decreasing, the macropore size increased and the number of pore throat decreased. When the internal phase volume fraction was 20%, the number of macro- and interconnected pores decreased significantly, while the interconnected pore still existed. This phenomenon indicated that the closely packed pore was not the only reason for forming the interconnected pore. Actually, MMT particles and the surfactant will both absorb onto the O/W interface, and the interconnected pore can also be formed at the interface stabilized by the surfactant (Zhu et al., 2016b). The macropore size and pore throat void size distribution of the prepared macroporous monoliths with variation of oil phase volume fraction were shown in Figure S1.

#### FTIR Analysis

**Figure 6:**
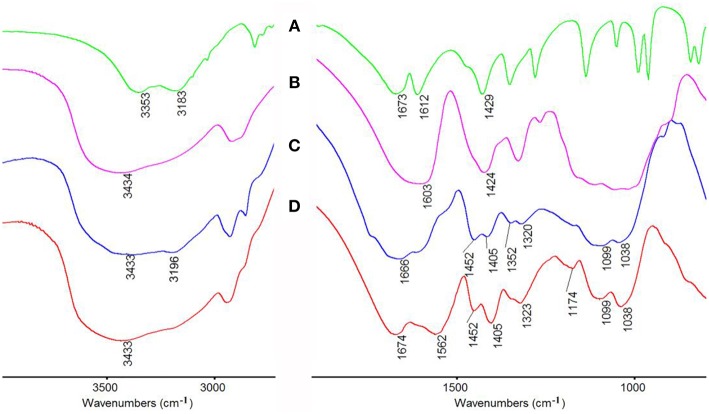
FTIR spectra of **(A)** AM, **(B)** CMC, CMC-*g*-PAM/MMT **(C)** before and **(D)** after hydrolysis reaction.

The macroporous monoliths were synthesized by free radical polymerization with APS as the initiator. The sulfate anion radicals formed from the decomposition of APS under 65°C, and the –OH groups of CMC was activated and formed the macro-radicals. Later, the macro-radicals triggered the double bonds of AM and *in situ* initiated the grafting reaction of the vinyl monomers onto the CMC in the presence of cross-linker MBA. Figure 6 showed the FTIR spectra of (a) AM, (b) CMC, and CMC-*g*-PAM/MMT (c) before and after hydrolysis reaction (d). As can be seen, the band at 3,433 cm^−1^ in Figures 6C, D was attributed to the characteristic absorption peak of O–H and intermolecular hydrogen bonds of the polysaccharide. The narrow band of C = C stretching vibration at 1,612 cm^−1^ in the AM spectrum disappeared after polymerization (Figure 6C). Besides, the band at 1,673 cm^−1^ in the AM spectrum that was ascribed to the vibration of C = O of amide was merged with the strong band (1,603 cm^−1^) of carboxylate asymmetric vibration in the CMC spectrum and formed a strong and broad absorption band at 1,666 cm^−1^ in Figure 6C. After the hydrolysis of macroporous monoliths in the alkaline solution (Figure 6D), the absorption peak at 1,666 cm^−1^ weakened, and the asymmetric stretching vibration (1,562 cm^−1^) and symmetrical stretching (1,407 cm^−1^) vibration of -COO^−^ strengthened. All of these proved that AM had been grafted onto CMC and a majority of acid amide groups were transformed into carboxylate groups after the hydrolysis (Ghorai et al., 2014).

**Figure 7:**
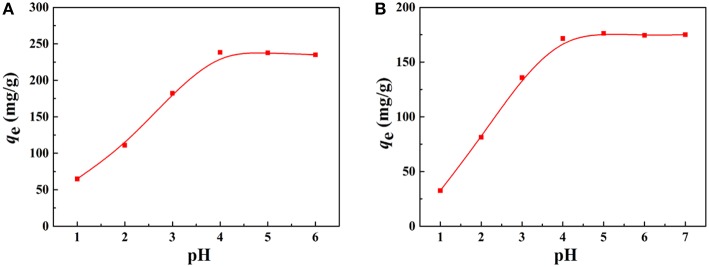
Effect of pH on the adsorption capacity of the macroporous monolith (Sample 6) for **(A)** Pb^2+^ and **(B)** Cd^2+^, respectively. Adsorption conditions: *C*_0_, 200 mg/L; dosage of adsorbent, 20 mg/25 mL.

### Evaluation of Adsorption Properties

#### Effect of pH on Adsorption for Pb^2+^ and Cd^2+^

**Figure 8:**
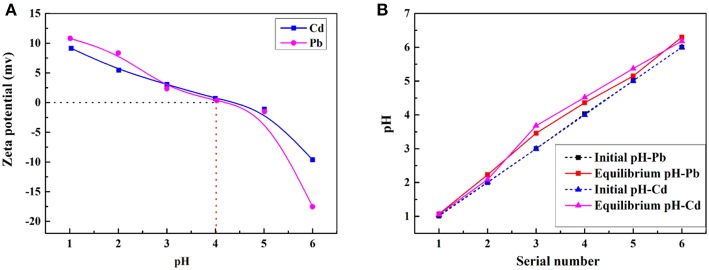
**(A)** Zeta potential of CMC-*g*-PAM/MMT at different pH values. Adsorption experiments: *C*_0_, 200 mg/L; t, 1 h; dosage of adsorbent, 50 mg/20 mL. **(B)** Initial and equilibrium pH of the solutions. Adsorption experiments: *C*_0_, 200 mg/L; t, 1 h; dosage of adsorbent, 20 mg/25 mL.

The adsorption property of adsorbents for metal ions depends on the charge of binding sites and the metal species present in the solution. Therefore, the pH values of the metal ion solution and isoelectric point (pH of zero point charge, pH_IEP_) of the prepared porous adsorbent (Sample 6) were determined to better understand the adsorption process. The pH_IEP_, which represents the net external charge on the surface of the adsorbent in solution (Chingombe et al., 2005), was found to be about 4.0 for Sample 6 (Figure 8A). The adsorption property of the macroporous monolithic adsorbent for the heavy metal ions was investigated in different pH solutions ranging from 1 to 6. As shown in Figure 7, it can be found that the adsorption of Pb^2+^ and Cd^2+^ increased quickly at pH 1–4 and then remained constant. Figure 8 showed that the surface of Sample 6 was positively charged at pH lower than pH_IEP_, while at pH > pH_IEP_, the surface of Sample 6 was negatively charged. Under acidic conditions, the adsorbents are usually protonized. When pH < 4.0, the protonation of the functional groups resulted in the adsorbent being positively charged and thus rejecting adsorption of Pb^2+^ and Cd^2+^, leading to lower adsorption capacity. As the pH increases, the protonation of the functional groups decreases, thus increasing the adsorption capacity. At pH > 4.0, the negatively charged adsorbent attracts the metal cations to increase adsorption capacity until all the active sites were occupied, and then the saturation adsorption was reached.

Here, it also should be noted that after the adsorption, the equilibrium pH values of the Pb^2+^ and Cd^2+^ solution were all higher than the initial pH (Figure 8B). Under acidic conditions, along with the adsorption process, the -COO^−^ and -CONH_2_ groups were protonated and converted to -COOH and -${\text{CONH}}_{3}^{+}$, which consumes H^+^ of the solution, thus increasing the pH. The above discussion indicated that the electrostatic and complexation interactions mainly contribute to the adsorption process (Sitko et al., 2016). Taking into account that the adsorbent had high adsorption capacity for metal ions at high pH, but metal ions will precipitate at excessive high pH, the next adsorption experiments were performed at natural pH.

#### Effect of Initial Concentration on Adsorption for Pb^2+^ and Cd^2+^

**Figure 9:**
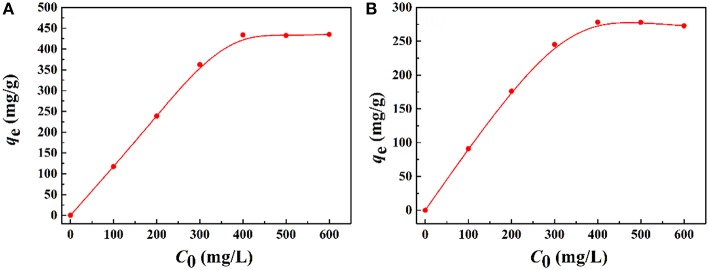
Effect of the initial **(A)** Pb^2+^ and **(B)** Cd^2+^ concentration on the adsorption capacity of macroporous monolith (Sample 6). Adsorption conditions: dosage of adsorbent, 20 mg/25 mL; pH, natural.

The saturation adsorption capacities of the macroporous monoliths for Pb^2+^ and Cd^2+^ were evaluated, and the results were shown in Figure 9. The increase in initial metal ions concentrations led to a sharp increase in adsorption capacities, and then the increasing trend became flat until adsorption saturation was reached. The reason was that the driving force at the solid–liquid interface improved with an increase in the initial metal ion concentration, which accelerated the diffusion of metal ions in the matrix and adsorbents. The maximum adsorption capacities of the macroporous monoliths were 456.05 mg/g for Pb^2+^ and 278.11 mg/g for Cd^2+^.

The adsorption process was analyzed by the Langmuir (Equation 2) and Freundlich isotherm models (Equation 3) (Gan et al., 2018), and the equations are shown as follows:

(2) $$C_{\text{e}}/\;q_{\text{e}}=1/(q_{\text{m}}\times b)+C_{\text{e}}/q_{\text{m}}$$

(3) $$Log\;q_{\text{e}}=logK+(1/n)log\;C_{\text{e}}$$

where *C*_e_ (mg/L) represents the equilibrium concentration of metal ions, and *q*_m_ and *q*_e_ are the amounts of metal ions adsorbed per unit mass of adsorbent at equilibrium state at any time (mg/g), respectively. *b* (L/mg) is the Langmuir constant related to the affinity of binding sites, *K* is the Freundlich constant related to adsorption capacity, and *n* represents the index of adsorption intensity or surface heterogeneity.

The isotherm parameters and linear correlation coefficients (*R*^2^) for the two models were calculated and summarized in Table S1. The adsorption capacities calculated by the Langmuir model for Pb^2+^ and Cd^2+^ were 464.91 and 315.82 mg/g, respectively. The calculated results were closer to the experiment value. Otherwise, all the adsorption isotherm data were better fitted to the Langmuir isotherm model (*R*^2^ > 0.99) than the Freundlich model. Thus, the Langmuir model was suitable to describe the adsorption process, which means that the binding sites distributed over the adsorbent surface was homogeneous and these binding sites had the same affinity for adsorption of a single molecular layer (Wen et al., 2012; Wang et al., 2013).

#### Effect of Contact Time on Adsorption for Pb^2+^ and Cd^2+^

**Figure 10:**
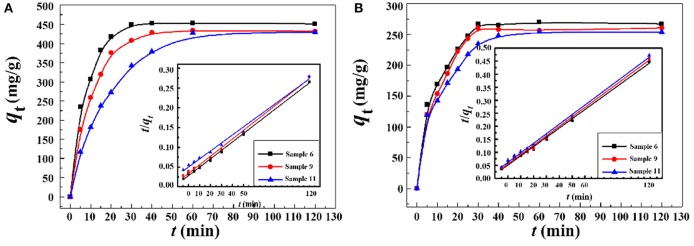
Adsorption kinetic curves of the macroporous monolith with various oil phase volume fraction for **(A)** Pb^2+^ and **(B)** Cd^2+^. Adsorption conditions: *C*_0_, 400 mg/L; dosage of adsorbent, 20 mg/25 mL; pH, natural.

The adsorption rate is a key parameter for practical applications. Thus, the effect of contact time on the adsorption behavior was investigated. Figure 10 showed the kinetic adsorption curves of the macroporous monoliths with different oil phase volume fraction for Pb^2+^ and Cd^2+^. The adsorption rate of the macroporous monoliths for the metal ions increased remarkably in the first 30 min for Pb^2+^ and Cd^2+^. In particular, Sample 6 and Sample 9 with well-interconnected pore structure showed fast adsorption rate. This was because the interconnected pores reduced the mass transfer resistance efficiently and exposed more carboxyl groups (as the binding sites for metal ions), which facilitate the accessibility of metal ions to the adsorbent. However, a large decrease in the adsorption rate was observed when the adsorbent with a relatively small number of macro- and interconnected pores and Sample 11 (prepared with 20% of internal phase volume) reached the equilibrium more than 1 h. The above facts proven that the structure of the adsorbents dramatically affected the adsorption kinetics of the macroporous monoliths for the metal ions. The high porosity and permeability of the adsorbent can improve the adsorption rate.

In order to examine the dynamic adsorption process, the adsorption data were fitted by pseudo-first-order (Equation 4) and pseudo-second-order (Equation 5) (Wu et al., 2018) kinetic models. The kinetic models' equations are expressed as follows:

(4) $$Log(q_{\text{e}}-q_{\text{t}})=logq_{\text{e}}-(k_{1}/2.303)t$$

(5) $$t/q_{t=}1/(k_{2}\times q_{\text{e}}^{2})+t/q_{\text{e}}$$

Here, *q*_t_ is the amount adsorbed for Pb^2+^ and Cd^2+^ at time *t* and *q*_e_ is the amount of metal ions by unit mass of adsorbents at equilibrium state. *k*_1_ (min^−1^) and *k*_2_ [g/(mg min)] are the adsorption rate constants of the pseudo-first-order and pseudo-second-order models, respectively. The corresponding adsorption kinetic parameters are listed in Tables S2, S3. As shown in Tables S2, S3, the linear correlation coefficients (*R*^2^) for the pseudo-second-order kinetic model were much higher than those for the pseudo-first-order kinetic model. Otherwise, the adsorption capacities calculated with the pseudo-second-order kinetic model (*q*_e,cal_) were much closer to the experiment values (*q*_e,exp_). Thus, the pseudo-second-order kinetic model fit the dynamic adsorption process well and it was controlled mainly by a chemical adsorption process (Zhou et al., 2014).

#### Regeneration and Reusability

**Figure 11:**
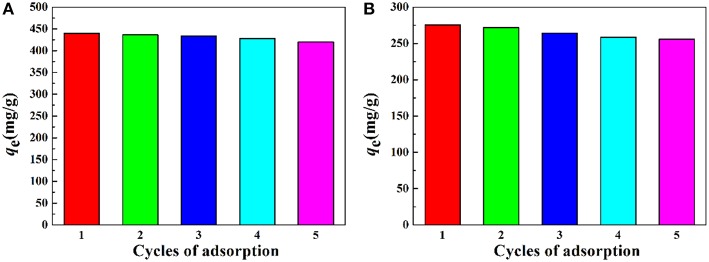
Adsorption capacity of the macroporous monolith (Sample 6) for **(A)** Pb^2+^ and **(B)** Cd^2+^ after regenerated five times. Adsorption conditions: *C*_0_, 400 mg/L; adsorbent dosage: 20 mg/25 mL; pH, natural.

**Figure 12:**
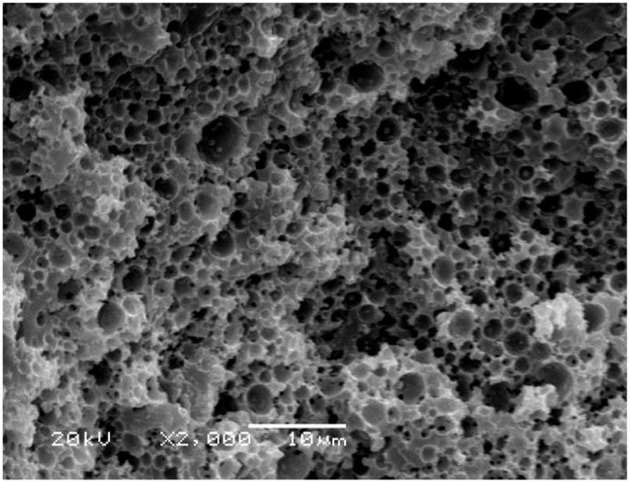
SEM of the macroporous CMC-*g*-PAM/MMT monolith after metal-adsorption (Sample 1).

The reusability of the as-prepared macroporous monolith was tested by repeating the adsorption–desorption cycle using 0.5 mol/L HCl as the desorbing agent and 0.5 mol/L NaOH as regeneration solution. As shown in Figure 11, the adsorption capacity had no obvious decrease with increasing the adsorption–desorption cycle. The regenerated monolith still showed high adsorption capabilities for Pb^2+^ and Cd^2+^ after being reused five times. Moreover, it should be noted that, after metal adsorption, the adsorbent still maintained a well-porous structure (Figure 12), and it also can be seen from Table 2 that the *q*_m_ values of the macroporous monolith prepared in this study were much higher than those of the adsorbents reported previously. All the adsorption dates indicated that the as-prepared macroporous monolith materials were an efficient adsorbent for heavy metal ions and had potential application in purification of wastewater.

**Table 2:** Comparison of adsorption capacities (*q*_m_, mg/g) of various adsorbents for Pb^2+^ and Cd^2+^.

**Adsorbent**	**Cd^**2+**^ (mg/g)**	**Pb^**2+**^ (mg/g)**	**References**
Tourmaline		108	Wang et al., 2011
Lignosulfonate–graphene oxide–polyaniline ternary nanocomposite		216	Yang et al., 2014
EDTA–graphene oxide		479	Madadrang et al., 2012
Fe_3_O_4_@DAPF core–shell ferromagnetic nanorods (CSFMNRs)		83	Venkateswarlu and Yoon, 2015
Thiocarbohydrazide cross-linked oxidized chitosan and poly(vinyl alcohol)		48	Ahmad et al., 2017
Magnetic cellulose nanocrystal/metal–organic framework composite (MCNC@Zn-BTC)		559	Wang et al., 2017a,b
Thermophilic *Geobacillus galactosidasius* sp. nov. loaded γ-Fe_2_O_3_ magnetic nanoparticle	37		Özdemir et al., 2016
Zr-based MOF-808 supported on polyacrylonitrile (PAN) nanofiber	225		Efome et al., 2018
TP–PS biocomposite hydrogels	194		Maity and Ray, 2017
Polyvinyl alcohol/polyacrylic acid double network gel	116	195	Chu et al., 2015
Polyampholyte	182	202	Copello et al., 2012
Thiosemicarbazide modified chitosan manlin	257	325	Li et al., 2016
Magnetic chelating resin (MIDA)	247	745	El-Bahy, 2018
MnO_2_ modification of biochar (BR)	14	128	Liang et al., 2017
CMC-*g*-PAM/MMT	278	456	This study

#### Adsorption Mechanism

**Figure 13:**
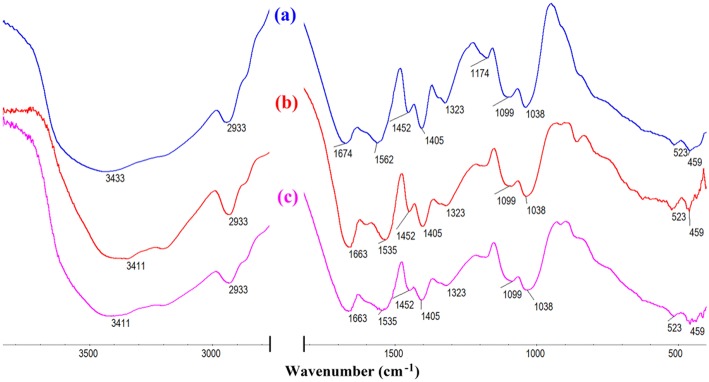
The FTIR spectra of CMC-*g*-PAM/MMT before **(a)** and after **(b)** Pb^2+^, **(c)** Cd^2+^ adsorption.

FTIR and XPS of CMC-*g*-PAM before and after adsorption of Pb^2+^ and Cd^2+^ would provide powerful evidence to clarify the adsorption mechanism of metal ions onto the functional groups of the as-prepared adsorbent. As can be seen from the FTIR spectra in Figure 13, after adsorption of Pb^2+^ or Cd^2+^, the strong absorption band at 3,434 cm^−1^ assigned to the O–H stretching vibration shifted to 3,411 cm^−1^, indicating the electrostatic interaction and H-bonding between -OH and Pb^2+^ or Cd^2+^. Besides, the band at 1,674 cm^−1^ ascribed to the vibration of C = O of carboxyl, and the asymmetric stretching vibration of -COO^−^ at 1,562 cm^−1^ shifted to 1,663 and 1,535 cm^−1^ after adsorption, which proved that the combination of carboxyl and metal ions reduced electron cloud density of oxygen atoms on carboxyl. According to the above, it can be confirmed that the carboxyl group and the hydroxyl group are the main force of the adsorbent to adsorb metal ions.

**Figure 14:**
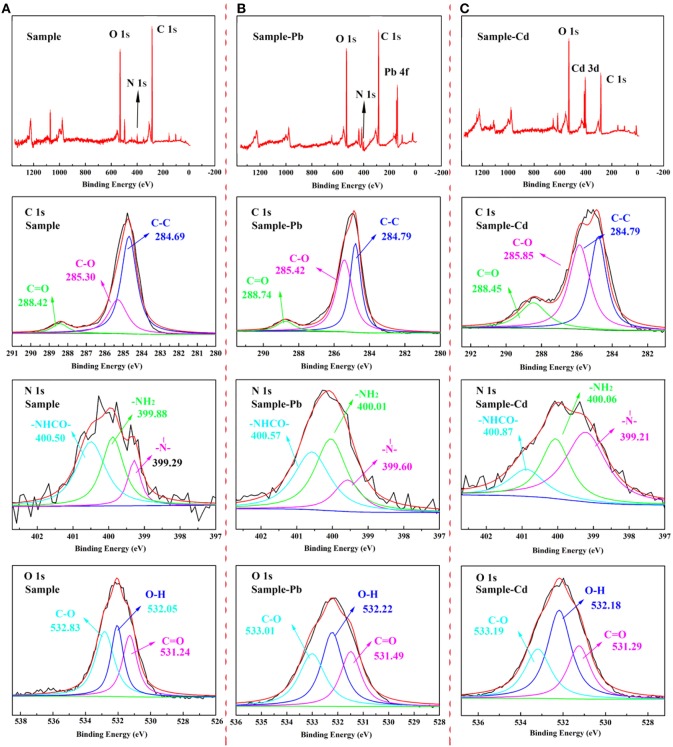
The XPS spectra of CMC-*g*-PAM/MMT before **(A)** and after **(B)** Pb^2+^, **(C)** Cd^2+^ adsorption.

The XPS spectra of CMC-*g*-PAM/MMT before and after adsorption were depicted in Figure 14, and the corresponding parameters were listed in Table 3. As the survey spectrum in Figures 14B,C showed, the appearance of Pb 4f and Cd 3d at 139.48 and 412.38 eV after adsorption revealed that the Pb^2+^ and Cd^2+^ were specifically adsorbed onto the prepared porous adsorbent. In the high-resolution XPS spectra of C 1s, there were also obvious changes in integral area ratio and binding energy after adsorbing Pb^2+^ and Cd^2+^. As listed in Table 3, the integral area ratios of C = O and C–O were both increased, while the integral area ratio of C–C decreased. The shift of binding energy can also be observed in the spectra, especially for the binding energy of C = O and C–O, which can be attributed to the decreased electron density of the adjacent O atoms, and thus the binding energy of the C atoms increased (Ma et al., 2019). The high-resolution O 1s spectra before adsorption presented three peaks at a binding energy of 532.83, 532.05, and 531.24 eV, which are assigned to the C–O, O–H, and C = O groups, respectively. After the adsorption of heavy metal ions, the binding energy increased; meanwhile, the integral area ratio of C = O and C–O decreased, and the integral area ratio of O–H increased. The shift of binding energy was attributed to the complexation between metal and -OH and -COO^−^, in which O atoms donate electrons to metal ions and the electron density toward O atoms in these groups decrease, thus increasing the binding energy of O 1s peaks. The above results demonstrate that the -COO^−^ and -OH groups in porous adsorbent participate in the adsorption of heavy metal ions, but in different ways (Luo et al., 2014). Otherwise, the weak signal peak of N 1s in the XPS spectra showed that there was only a slight amount of acylamino existing in the materials after hydrolysis. As the high-resolution XPS spectra of N 1s shown, the binding energy of -NH_2_ and -NHCO- increased slightly, while their integral area ratio decreased after the adsorption of heavy metal ions, demonstrating that the acylamino groups participated in the adsorption process. These results revealed the electrostatic attraction or coordination interaction between the functional groups and heavy metal ions, and the COO^−^ and -OH groups provide the main adsorption sites. The acylamino groups assisted in the adsorption process.

**Table 3:** Binding energy (eV) of CMC-*g*-PAM/MMT before and after adsorbed with Pb^2+^ and Cd^2+^.

**Element**		**Sample**		**Sample -Pb**		**Sample -Cd**	
		**BE(eV)**	**Percent (%)**	**BE (eV)**	**Percent (%)**	**BE (eV)**	**Percent (%)**
C1S	C–C	284.69	65.99	284.79	42.14	284.79	38.54
	C–O	285.30	27.44	285.42	50.32	285.85	44.20
	C = O	288.42	6.57	288.74	7.54	288.46	17.25
N1S	$\overset{|}{-\text{N}-}$	399.29	14.75	399.57	16.36	399.21	51.51
	-NH2	399.88	37.81	400.04	43.62	400.06	31.23
	-NHCO-	400.50	47.44	400.57	40.02	400.87	17.27
O1S	C = O	531.29	29.95	531.49	27.31	531.24	26.12
	O–H	532.05	29.69	532.22	38.80	532.18	45.92
	C–O	532.83	40.35	533.01	33.88	533.18	27.96

## Conclusions

A series of novel macroporous monolithic adsorbents had been successfully prepared by polymerization of Pickering MIPEs with natural MMT and T-20 as the stabilizer and the non-toxic and eco-friendly flaxseed oil used in disperse phase. The structure of the macroporous monolith can be adjusted by changing the volume of oil phase and the content of MMT and T-20. The synergistic effects of MMT and T-20 were favorable to create the macroporous monolith materials with a closed-cell or highly interconnected macroporous structure. The prepared macroporous monoliths showed fast adsorption kinetics for Pb^2+^ and Cd^2+^, and the adsorption equilibrium can be reached as fast as 30 min. Otherwise, the adsorption rate of the adsorbent can be improved by increasing the connectivity and porosity of the monolith. The adsorption capacities of the adsorbent for Pb^2+^ and Cd^2+^ were 456.05 and 278.11 mg/g, respectively. In addition, the macroporous monolith exhibited excellent reusability. After five adsorption–desorption cycles, the regenerated monolith still had good absorbability and maintained a well-porous structure. As a whole, we provided a new approach to fabricating macroporous monoliths that are highly efficient and recyclable using a type of eco-friendly Pickering MIPE. The prepared monoliths can be used as efficient adsorbent for the removal of heavy metals from wastewater.

## Data Availability

The datasets generated for this study are available on request to the corresponding author.

## Author Contributions

FW and YZ contributed to the experiment process and data analysis, wrote the paper, and created all the figures. HX contributed to the design of the experiment, data analysis, and revision of the paper. AW contributed to the experiment design, data analysis, and revision of the paper.

### Conflict of Interest Statement

The authors declare that the research was conducted in the absence of any commercial or financial relationships that could be construed as a potential conflict of interest.
