# Supplementary material for: Preparation of Carboxymethyl Cellulose-Based Macroporous Adsorbent by Eco-Friendly Pickering-MIPEs Template for Fast Removal of Pb2+ and Cd2+
Source: Front Chem. 2019 Sep 10;7:603. doi: 10.3389/fchem.2019.00603 (PMC6746836; doi:10.3389/fchem.2019.00603)
Supplement: Supplementary file 1 [file Table_1.DOC]

**Supplementary materials**

**Preparation of carboxymethyl cellulose-based macroporous adsorbent by eco-friendly Pickering-MIPEs template for fast removal of Pb^2+^ and Cd^2+^**

**Feng Wang^1,2^,** **Yongfeng Zhu^1^,** **Hui Xu^3^, Aiqin Wang^1^,***

^1^Key Laboratory of Clay Mineral Applied Research of Gansu Province, Center of Eco-material and Green Chemistry, Lanzhou Institute of Chemical Physics, Chinese Academy of Sciences, Lanzhou, 730000, P.R. China

^2^College of Petroleum and Chemical Engineering，Beibu Gulf University，Qinzhou 535011, P.R. China;

^3^Department of Chemical Engineering, College of Petrochemical Engineering, Lanzhou University of Technology, Lanzhou, 730050, PR China

*** Correspondence:**

Corresponding author.

E-mail:[aqwang@licp.cas.cn](mailto:aqwang@licp.cas.cn)(A.Q. WANG)


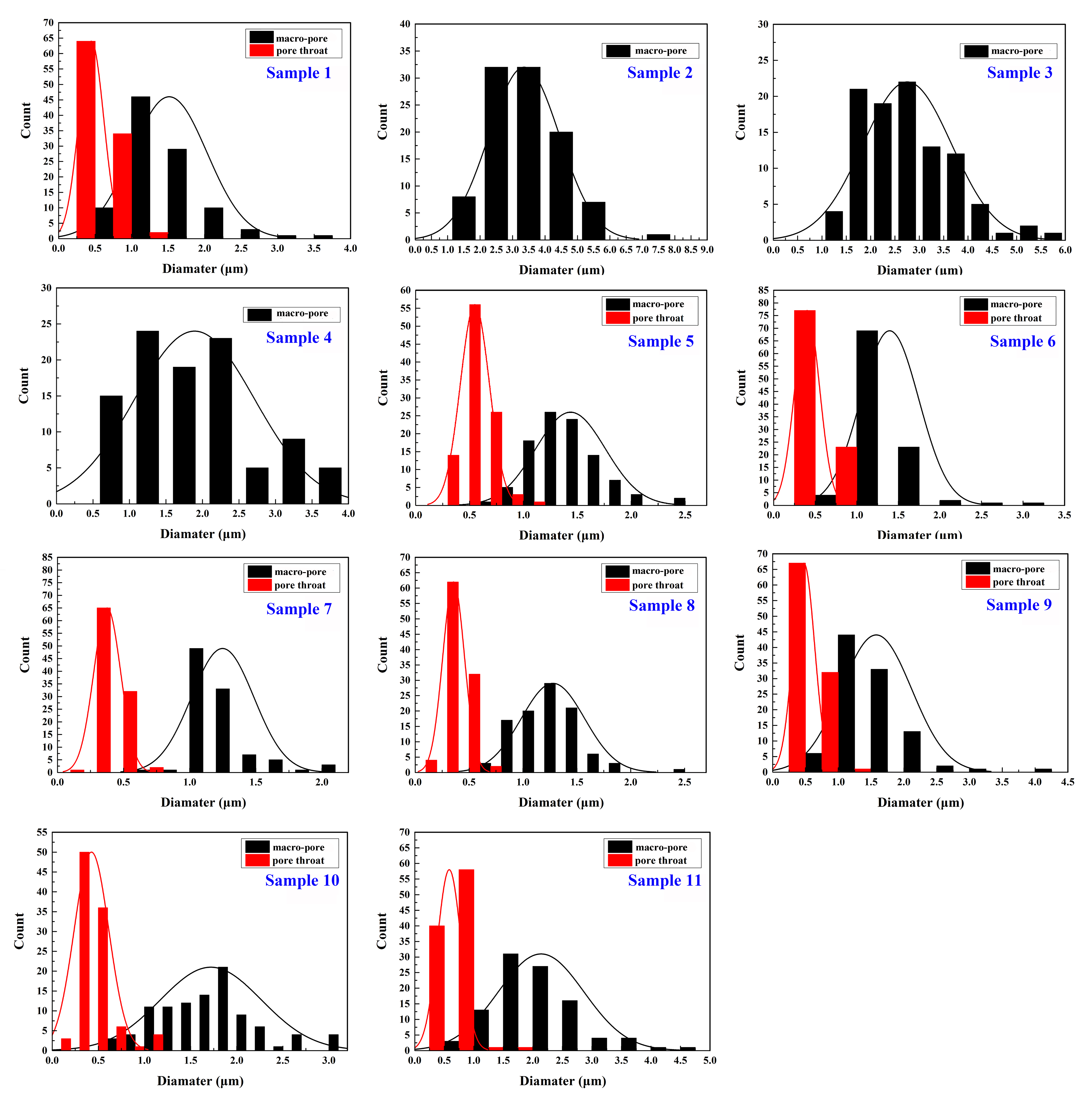


Fig. S1 The pore (macroporous and pore throat) size distribution of the macroporous CMC-*g*-PAM/MMT prepared in different conditions.

Table S1 Adsorption isotherm constants for the adsorption of Pb^2+^ and Cd^2+^ onto macroporous CMC-*g*-PAM/MMT monoliths.

| ***Langmuir model*** | | | | | | | | | | | | | | |
| --- | --- | --- | --- | --- | --- | --- | --- | --- | --- | --- | --- | --- | --- | --- |
| Heavy mental | | **Pb^2+^** | | | | | |  | | **Cd^2+^** | | | | |
| Samples | *q*_e, exp_  (mg/g) | | *q*_m_  (mg/g) | | *b*  (L/mg) | | R^2^ | |  | | *q*_e, exp_  (mg/g) | *q*_m_  (mg/g) | *b*  (L/mg) | R^2^ |
| Sample 6 | 451.42 | | 464.91 | | 0.1783 | | 0.9977 | |  | | 277.83 | 315.82 | 0.0227 | 0.9958 |
| ***Freundlich model*** | | | | | | | | | | | | | | |
| Samples | K | | | *n* | | R^2^ | | |  | K | | *n* | | R^2^ |
| Sample 6 | 140.01 | | | 4.0350 | | 0.5054 | | |  | 35.40 | | 2.6526 | | 0.6781 |

Table S2 Adsorption kinetic parameters for adsorption of Pb^2+^ onto the macroporous CMC-*g*-PAM/MMT monoliths.

|  | **Pb^2+^** | | | | | | |  |  |
| --- | --- | --- | --- | --- | --- | --- | --- | --- | --- |
|  | **Pseudo-first-order equation** | | |  | **Pseudo-second-order equation** | | | | |
| Samples | *q*_e,cal_  (mg/g) | *K*_1_×10^-2^  (min^-1^) | *R*^2^ |  | *q*_e,cal_  (mg/g) | *K*_2_×10^-4^  (g/mg min) | *R*^2^ |  | *q*_e,exp_  (mg/g) |
| Sample 6 | 97.07 | 4.54 | 0.4344 |  | 468.94 | 6.65 | 0.9979 |  | 453.46 |
| Sample 9 | 190.31 | 4.77 | 0.7836 |  | 459.77 | 3.87 | 0.9962 |  | 433.91 |
| Sample 11 | 922.72 | 11.24 | 0.8004 |  | 494.12 | 1.36 | 0.9928 |  | 428.89 |

Table S3 Adsorption kinetic parameters for adsorption of Cd^2+^ onto the macroporous CMC-*g*-PAM/MMT monoliths.

|  | **Cd^2+^** | | | | | | |  |  |
| --- | --- | --- | --- | --- | --- | --- | --- | --- | --- |
|  | **Pseudo-first-order equation** | | |  | **Pseudo-second-order equation** | | | | |
| Samples | *q*_e,cal_  (mg/g) | *K*_1_×10^-2^  (min^-1^) | *R*^2^ |  | *q*_e,cal_  (mg/g) | *K*_2_×10^-4^  (g/mg min) | *R*^2^ |  | *q*_e,exp_  (mg/g) |
| Sample 6 | 80.36 | 3.63 | 0.5054 |  | 281.47 | 8.09 | 0.9966 |  | 270.28 |
| Sample 9 | 199.50 | 8.31 | 0.8480 |  | 275.37 | 7.26 | 0.9957 |  | 260.72 |
| Sample 11 | 187.55 | 6.65 | 0.97447 |  | 272.55 | 5.44 | 0.9961 |  | 254.28 |
